# *N*,*N*′-Bis[4-(trifluoro­meth­yl)phen­yl]pyridine-2,6-dicarboxamide

**DOI:** 10.1107/S1600536810001753

**Published:** 2010-01-20

**Authors:** Le Chen, Hongwu He, Hao Peng

**Affiliations:** a Key Laboratory of Pesticide and Chemical Biology, College of Chemistry, Central China Normal University, Wuhan 430079, People’s Republic of China.

## Abstract

In the title mol­ecule, C_21_H_13_F_6_N_3_O_2_, the pyridine ring forms dihedral angles of 1.7 (1) and 5.2 (1)° with the two benzene rings. In the crystal structure, inter­molecular N—H⋯O hydrogen bonds and π⋯π inter­actions [centroid–centroid distance of 3.628 (3) Å between aromatic rings] link mol­ecules into stacks along the *c* axis. The two trifluoro­methyl groups are each rotationally disordered between two orientations, with occupancy ratios of 0.58 (1):0.42 (1) and 0.55 (1):0.45 (1).

## Related literature

For the synthesis and biological activity of acyl thio­urea derivatives, see: Duan *et al.* (2003 ▶); Li *et al.* (2007 ▶).
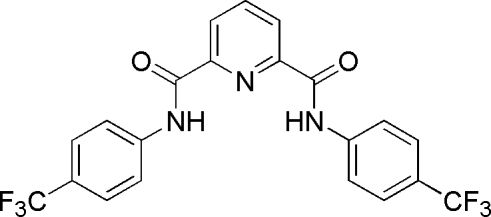


## Experimental

### 

#### Crystal data

C_21_H_13_F_6_N_3_O_2_
*M* *_r_* = 453.34
Monoclinic, 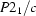

*a* = 9.8308 (10) Å
*b* = 23.787 (3) Å
*c* = 8.9577 (10) Å
β = 109.474 (2)°
*V* = 1974.8 (4) Å^3^
*Z* = 4
Mo *K*α radiation
μ = 0.14 mm^−1^
*T* = 298 K
0.20 × 0.12 × 0.10 mm

#### Data collection

Bruker SMART CCD area-detector diffractometer
12033 measured reflections
3653 independent reflections
3138 reflections with *I* > 2σ(*I*)
*R*_int_ = 0.043

#### Refinement

*R*[*F* ^2^ > 2σ(*F* ^2^)] = 0.078
*wR*(*F* ^2^) = 0.181
*S* = 1.17
3653 reflections
351 parameters
96 restraints
H atoms treated by a mixture of independent and constrained refinement
Δρ_max_ = 0.32 e Å^−3^
Δρ_min_ = −0.25 e Å^−3^

### 

Data collection: *SMART* (Bruker, 2001 ▶); cell refinement: *SAINT* (Bruker, 2001 ▶); data reduction: *SAINT*; program(s) used to solve structure: *SHELXS97* (Sheldrick, 2008 ▶); program(s) used to refine structure: *SHELXL97* (Sheldrick, 2008 ▶); molecular graphics: *PLATON* (Spek, 2009 ▶); software used to prepare material for publication: *PLATON*.

## Supplementary Material

Crystal structure: contains datablocks global, I. DOI: 10.1107/S1600536810001753/cv2671sup1.cif

Structure factors: contains datablocks I. DOI: 10.1107/S1600536810001753/cv2671Isup2.hkl

Additional supplementary materials:  crystallographic information; 3D view; checkCIF report

## Figures and Tables

**Table 1:** Hydrogen-bond geometry (Å, °)

*D*—H⋯*A*	*D*—H	H⋯*A*	*D*⋯*A*	*D*—H⋯*A*
N2—H2*A*⋯O1^i^	0.84 (3)	2.58 (3)	3.303 (4)	144 (3)
N3—H3*A*⋯O2^ii^	0.85 (4)	2.36 (4)	3.051 (3)	139 (3)

Symmetry codes: (i) 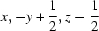; (ii) 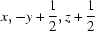.

**Fig. 1.**
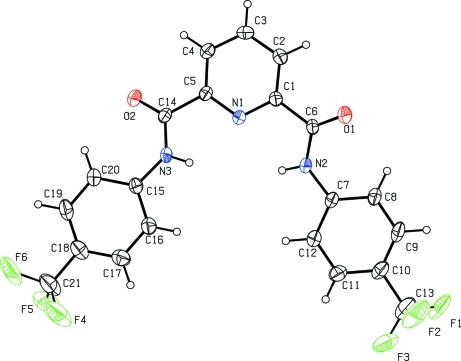
Molecular structure of (I), showing the labeling scheme with 50% probability displacement ellipsoids. Only major parts of disordered fluorine atoms are shown.

**Fig. 2.**
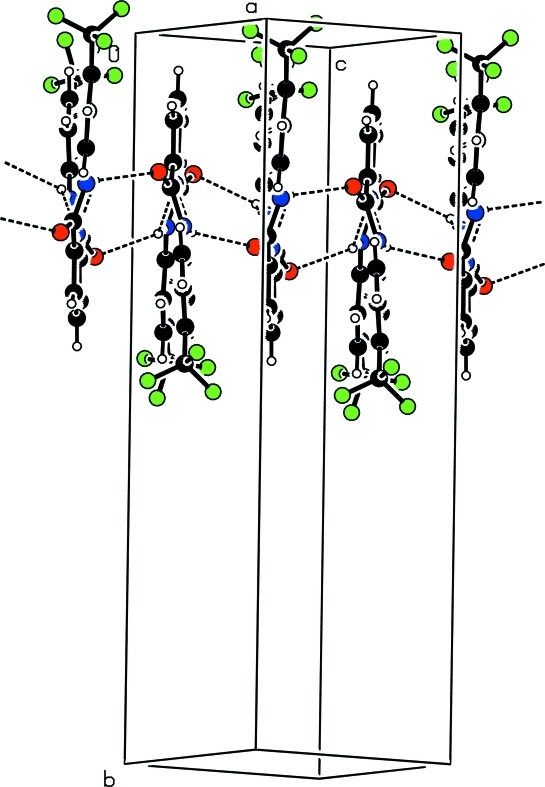
Part of the crystal packing, showing the intermolecular hydrogen bonds as dashed lines.

## supplementary crystallographic information

### Comment

Acyl thiourea derivatives have attracted considerable attention from chemists and biologists because of their wide range of biological activities and potential therapeutic value (Duan *et al.*, 2003; Li *et al.*, 2007). In our research work aimed at searching for novel agrochemicals, we attempted to synthesize the pyridine-2,6-dicarbonyl thiourea derivatives,and the title compound, (I), was obtained as byproduct. Here we report its crystal structure.

In (I) (Fig.1), the central pyridine ring makes the dihedral angles of 1.7 (1) and 5.2 (1)° with the two benzene rings, respectively. In the crystal structure, intermolecular N—H···O hydrogen bonds (Table 1) and π–π interactions proved by short distance of 3.628 (3) Å between the centroids of aromatic rings link molecules into stacks along axis *c*.

### Experimental

Pyridine-2,6-dicarbonyl chloride (II) was prepared according to the literature procedures (Duan *et al.*, 2003). To a solution of (II) (5 mmol) in dichloromethane (30 ml) was added KSCN (15 mmol) and PEG-400 (0.2 g). The mixture was stirred at room temperature for 2 h, then 4-trifluoroaniline (10 mmol) was added and the suspension was stirred for 1 h. The mixture was filtered and the precipitate was washed with a little ethanol. The solvent was removed under reduced pressure and the residue was purified by recrystallization from DMF and water (20:1,*v*/*v*). Then recrystallization from actone over a period of one week gave colourless crystals of (I).

### Refinement

C-bound H atoms were geometrically positioned (C—H 0.93 Å) and refined as riding, with *U*_iso_(H) =1.2*U*_eq_(C). H atoms bonded to N atoms were located at the difference map, and refined with bond restrains N—H = 0.84 (3) Å, and with constrains of *U*_iso_(H) = 1.2*U*_eq_(N). Two trifluoromethyl groups were treated as rotationally disordered between two orientations each with the ratios refined to 0.58 (1):0.42 (1) and 0.55 (1):0.45 (1), respectively.

### Crystal data

**Table:**

C_21_H_13_F_6_N_3_O_2_	*F*(000) = 920
*M**_r_* = 453.34	*D*_x_ = 1.525 Mg m^−^^3^
Monoclinic, *P*2_1_/*c*	Mo *K*α radiation, λ = 0.71073 Å
Hall symbol: -P 2ybc	Cell parameters from 3607 reflections
*a* = 9.8308 (10) Å	θ = 2.4–25.0°
*b* = 23.787 (3) Å	µ = 0.14 mm^−^^1^
*c* = 8.9577 (10) Å	*T* = 298 K
β = 109.474 (2)°	Block, colourless
*V* = 1974.8 (4) Å^3^	0.20 × 0.12 × 0.10 mm
*Z* = 4	

### Data collection

**Table:**

Bruker SMART CCD area-detector diffractometer	3138 reflections with *I* > 2σ(*I*)
Radiation source: fine-focus sealed tube	*R*_int_ = 0.043
graphite	θ_max_ = 25.5°, θ_min_ = 2.2°
phi and ω scans	*h* = −11→11
12033 measured reflections	*k* = −28→22
3653 independent reflections	*l* = −10→10

### Refinement

**Table:**

Refinement on *F*^2^	Primary atom site location: structure-invariant direct methods
Least-squares matrix: full	Secondary atom site location: difference Fourier map
*R*[*F*^2^ > 2σ(*F*^2^)] = 0.078	Hydrogen site location: inferred from neighbouring sites
*wR*(*F*^2^) = 0.181	H atoms treated by a mixture of independent and constrained refinement
*S* = 1.17	*w* = 1/[σ^2^(*F*_o_^2^) + (0.0634*P*)^2^ + 1.4024*P*] where *P* = (*F*_o_^2^ + 2*F*_c_^2^)/3
3653 reflections	(Δ/σ)_max_ = 0.001
351 parameters	Δρ_max_ = 0.32 e Å^−^^3^
96 restraints	Δρ_min_ = −0.25 e Å^−^^3^

### Special details

**Table:**

Geometry. All e.s.d.'s (except the e.s.d. in the dihedral angle between two l.s. planes) are estimated using the full covariance matrix. The cell e.s.d.'s are taken into account individually in the estimation of e.s.d.'s in distances, angles and torsion angles; correlations between e.s.d.'s in cell parameters are only used when they are defined by crystal symmetry. An approximate (isotropic) treatment of cell e.s.d.'s is used for estimating e.s.d.'s involving l.s. planes.
Refinement. Refinement of *F*^2^ against ALL reflections. The weighted *R*-factor *wR* and goodness of fit *S* are based on *F*^2^, conventional *R*-factors *R* are based on *F*, with *F* set to zero for negative *F*^2^. The threshold expression of *F*^2^ > σ(*F*^2^) is used only for calculating *R*-factors(gt) *etc*. and is not relevant to the choice of reflections for refinement. *R*-factors based on *F*^2^ are statistically about twice as large as those based on *F*, and *R*- factors based on ALL data will be even larger.

### Fractional atomic coordinates and isotropic or equivalent isotropic displacement parameters (Å^2^)

**Table:**

	*x*	*y*	*z*	*U*_iso_*/*U*_eq_	Occ. (<1)
C1	0.1924 (3)	0.17950 (12)	0.0978 (3)	0.0384 (7)	
C2	0.1982 (4)	0.12169 (14)	0.1004 (4)	0.0491 (8)	
H2	0.1447	0.1009	0.1492	0.059*	
C3	0.2849 (4)	0.09535 (14)	0.0289 (4)	0.0557 (9)	
H3	0.2902	0.0563	0.0282	0.067*	
C4	0.3637 (3)	0.12687 (13)	−0.0413 (4)	0.0461 (8)	
H4	0.4218	0.1097	−0.0914	0.055*	
C5	0.3546 (3)	0.18462 (12)	−0.0356 (3)	0.0356 (6)	
C6	0.0990 (3)	0.21071 (13)	0.1728 (3)	0.0400 (7)	
C7	−0.0025 (3)	0.30625 (13)	0.1730 (3)	0.0394 (7)	
C8	−0.1031 (3)	0.29432 (15)	0.2475 (4)	0.0498 (8)	
H8	−0.1244	0.2573	0.2643	0.060*	
C9	−0.1704 (3)	0.33774 (17)	0.2959 (4)	0.0581 (9)	
H9	−0.2373	0.3298	0.3458	0.070*	
C10	−0.1409 (3)	0.39243 (16)	0.2721 (4)	0.0565 (9)	
C11	−0.0435 (4)	0.40445 (15)	0.1948 (5)	0.0620 (10)	
H11	−0.0236	0.4416	0.1772	0.074*	
C12	0.0234 (4)	0.36158 (14)	0.1444 (4)	0.0535 (9)	
H12	0.0869	0.3698	0.0904	0.064*	
C13	−0.2060 (4)	0.4394 (2)	0.3348 (5)	0.0853 (14)	
C14	0.4430 (3)	0.22150 (13)	−0.1048 (3)	0.0377 (7)	
C15	0.5242 (3)	0.32122 (13)	−0.0892 (3)	0.0400 (7)	
C16	0.4878 (4)	0.37330 (15)	−0.0475 (4)	0.0564 (9)	
H16	0.4215	0.3760	0.0057	0.068*	
C17	0.5477 (4)	0.42116 (16)	−0.0833 (5)	0.0661 (10)	
H17	0.5200	0.4561	−0.0567	0.079*	
C18	0.6488 (4)	0.41772 (16)	−0.1585 (4)	0.0599 (10)	
C19	0.6873 (4)	0.36594 (17)	−0.1989 (4)	0.0620 (10)	
H19	0.7560	0.3635	−0.2492	0.074*	
C20	0.6258 (3)	0.31734 (15)	−0.1662 (4)	0.0491 (8)	
H20	0.6518	0.2825	−0.1952	0.059*	
C21	0.7100 (6)	0.4701 (2)	−0.2000 (5)	0.0930 (16)	
N1	0.2702 (2)	0.21094 (10)	0.0320 (3)	0.0357 (6)	
N2	0.0727 (3)	0.26423 (11)	0.1232 (3)	0.0430 (6)	
H2A	0.110 (4)	0.2753 (14)	0.057 (4)	0.052*	
N3	0.4573 (3)	0.27437 (11)	−0.0487 (3)	0.0442 (7)	
H3A	0.420 (4)	0.2803 (14)	0.022 (4)	0.053*	
O1	0.0553 (3)	0.18852 (10)	0.2706 (3)	0.0663 (7)	
O2	0.4942 (3)	0.20366 (10)	−0.2017 (3)	0.0566 (7)	
F1	−0.3471 (7)	0.4321 (5)	0.3055 (10)	0.104 (3)	0.55 (1)
F2	−0.1415 (10)	0.4449 (5)	0.4902 (8)	0.130 (4)	0.55 (1)
F3	−0.1944 (10)	0.4899 (3)	0.2690 (12)	0.127 (4)	0.55 (1)
F4	0.7508 (11)	0.5071 (4)	−0.0789 (9)	0.143 (4)	0.58 (1)
F5	0.6273 (14)	0.4946 (5)	−0.3320 (9)	0.122 (4)	0.58 (1)
F6	0.8385 (8)	0.4579 (5)	−0.2243 (14)	0.133 (4)	0.58 (1)
F4'	0.8527 (8)	0.4711 (6)	−0.1667 (18)	0.119 (5)	0.45 (1)
F1'	−0.3145 (10)	0.4220 (6)	0.3872 (14)	0.130 (5)	0.45 (1)
F5'	0.6697 (11)	0.5178 (4)	−0.1386 (12)	0.109 (3)	0.45 (1)
F3'	−0.1123 (11)	0.4662 (5)	0.4586 (11)	0.088 (3)	0.42 (1)
F2'	−0.2616 (11)	0.4801 (5)	0.2255 (12)	0.110 (4)	0.42 (1)
F6'	0.6506 (17)	0.4783 (6)	−0.3602 (9)	0.102 (4)	0.42 (1)

### Atomic displacement parameters (Å^2^)

**Table:**

	*U*^11^	*U*^22^	*U*^33^	*U*^12^	*U*^13^	*U*^23^
C1	0.0394 (16)	0.0387 (17)	0.0408 (16)	−0.0035 (12)	0.0182 (13)	−0.0019 (13)
C2	0.0565 (19)	0.0434 (19)	0.0562 (19)	−0.0060 (15)	0.0305 (16)	−0.0004 (15)
C3	0.073 (2)	0.0319 (17)	0.072 (2)	0.0011 (15)	0.038 (2)	−0.0021 (16)
C4	0.0534 (18)	0.0430 (18)	0.0489 (18)	0.0050 (14)	0.0265 (15)	−0.0026 (14)
C5	0.0329 (14)	0.0392 (16)	0.0366 (15)	0.0050 (12)	0.0141 (12)	0.0006 (12)
C6	0.0395 (16)	0.0422 (18)	0.0456 (17)	−0.0055 (13)	0.0240 (14)	−0.0025 (13)
C7	0.0328 (15)	0.0467 (18)	0.0411 (16)	0.0030 (13)	0.0156 (13)	−0.0040 (13)
C8	0.0390 (17)	0.055 (2)	0.063 (2)	0.0000 (15)	0.0275 (16)	−0.0046 (16)
C9	0.0402 (18)	0.079 (3)	0.065 (2)	0.0021 (17)	0.0302 (17)	−0.0110 (19)
C10	0.0404 (18)	0.064 (2)	0.066 (2)	0.0063 (16)	0.0181 (16)	−0.0184 (18)
C11	0.053 (2)	0.048 (2)	0.089 (3)	0.0048 (16)	0.029 (2)	−0.0085 (19)
C12	0.0492 (19)	0.050 (2)	0.071 (2)	0.0047 (15)	0.0340 (17)	0.0023 (17)
C13	0.063 (3)	0.097 (4)	0.100 (4)	0.010 (3)	0.033 (3)	−0.036 (3)
C14	0.0383 (15)	0.0421 (17)	0.0366 (15)	0.0093 (12)	0.0176 (13)	0.0027 (13)
C15	0.0410 (16)	0.0450 (18)	0.0356 (15)	−0.0033 (13)	0.0148 (13)	0.0016 (13)
C16	0.065 (2)	0.052 (2)	0.064 (2)	−0.0091 (17)	0.0378 (19)	−0.0066 (17)
C17	0.084 (3)	0.047 (2)	0.076 (3)	−0.0112 (19)	0.039 (2)	−0.0073 (18)
C18	0.069 (2)	0.060 (2)	0.051 (2)	−0.0187 (18)	0.0202 (18)	0.0026 (17)
C19	0.062 (2)	0.077 (3)	0.059 (2)	−0.0150 (19)	0.0363 (19)	0.0027 (19)
C20	0.0469 (18)	0.054 (2)	0.0533 (19)	−0.0017 (15)	0.0264 (16)	0.0008 (16)
C21	0.127 (5)	0.078 (4)	0.087 (4)	−0.039 (3)	0.053 (4)	0.003 (3)
N1	0.0353 (12)	0.0357 (13)	0.0395 (13)	0.0021 (10)	0.0172 (10)	0.0003 (10)
N2	0.0471 (15)	0.0428 (16)	0.0516 (15)	0.0024 (11)	0.0330 (13)	0.0022 (12)
N3	0.0548 (16)	0.0436 (15)	0.0489 (15)	−0.0040 (12)	0.0371 (13)	−0.0027 (12)
O1	0.0879 (18)	0.0535 (15)	0.0839 (18)	0.0034 (13)	0.0640 (16)	0.0105 (13)
O2	0.0733 (16)	0.0520 (14)	0.0635 (15)	0.0044 (12)	0.0482 (13)	−0.0026 (11)
F1	0.063 (4)	0.126 (6)	0.131 (6)	0.029 (4)	0.044 (4)	−0.042 (5)
F2	0.124 (7)	0.159 (10)	0.107 (5)	0.023 (5)	0.040 (5)	−0.073 (5)
F3	0.128 (8)	0.071 (4)	0.203 (11)	0.015 (5)	0.084 (7)	−0.041 (6)
F4	0.209 (10)	0.102 (6)	0.134 (6)	−0.089 (6)	0.078 (7)	−0.024 (5)
F5	0.175 (7)	0.070 (7)	0.137 (7)	−0.004 (4)	0.072 (6)	0.036 (5)
F6	0.147 (7)	0.129 (8)	0.147 (8)	−0.095 (5)	0.081 (5)	−0.024 (5)
F4'	0.115 (7)	0.088 (7)	0.157 (11)	−0.060 (5)	0.049 (6)	−0.004 (7)
F1'	0.091 (7)	0.134 (9)	0.197 (12)	−0.011 (6)	0.092 (7)	−0.084 (9)
F5'	0.168 (9)	0.055 (4)	0.127 (7)	−0.043 (5)	0.082 (7)	−0.019 (5)
F3'	0.071 (4)	0.078 (6)	0.111 (7)	0.003 (4)	0.024 (5)	−0.050 (4)
F2'	0.097 (8)	0.094 (8)	0.118 (6)	0.052 (6)	0.007 (6)	−0.025 (6)
F6'	0.171 (10)	0.067 (8)	0.090 (6)	−0.008 (5)	0.075 (6)	0.022 (4)

### Geometric parameters (Å, °)

**Table:**

C1—N1	1.339 (4)	C13—F3'	1.342 (8)
C1—C2	1.376 (4)	C13—F2'	1.355 (8)
C1—C6	1.502 (4)	C13—F3	1.358 (7)
C2—C3	1.376 (4)	C13—F1'	1.365 (8)
C2—H2	0.9300	C14—O2	1.216 (3)
C3—C4	1.372 (4)	C14—N3	1.344 (4)
C3—H3	0.9300	C15—C16	1.375 (5)
C4—C5	1.379 (4)	C15—C20	1.393 (4)
C4—H4	0.9300	C15—N3	1.402 (4)
C5—N1	1.335 (3)	C16—C17	1.368 (5)
C5—C14	1.506 (4)	C16—H16	0.9300
C6—O1	1.217 (3)	C17—C18	1.377 (5)
C6—N2	1.345 (4)	C17—H17	0.9300
C7—C12	1.381 (5)	C18—C19	1.372 (5)
C7—C8	1.394 (4)	C18—C21	1.484 (5)
C7—N2	1.402 (4)	C19—C20	1.380 (5)
C8—C9	1.373 (5)	C19—H19	0.9300
C8—H8	0.9300	C20—H20	0.9300
C9—C10	1.365 (5)	C21—F5	1.326 (8)
C9—H9	0.9300	C21—F4'	1.334 (8)
C10—C11	1.386 (5)	C21—F4	1.350 (7)
C10—C13	1.488 (5)	C21—F6'	1.371 (8)
C11—C12	1.369 (5)	C21—F5'	1.375 (8)
C11—H11	0.9300	C21—F6	1.383 (8)
C12—H12	0.9300	N2—H2A	0.84 (3)
C13—F2	1.330 (7)	N3—H3A	0.85 (4)
C13—F1	1.335 (7)		
			
N1—C1—C2	122.6 (3)	F3'—C13—C10	113.9 (6)
N1—C1—C6	116.4 (2)	F2'—C13—C10	112.7 (7)
C2—C1—C6	120.9 (3)	F3—C13—C10	113.1 (6)
C1—C2—C3	118.4 (3)	F1'—C13—C10	112.7 (7)
C1—C2—H2	120.8	O2—C14—N3	125.2 (3)
C3—C2—H2	120.8	O2—C14—C5	121.6 (3)
C4—C3—C2	119.8 (3)	N3—C14—C5	113.2 (2)
C4—C3—H3	120.1	C16—C15—C20	119.2 (3)
C2—C3—H3	120.1	C16—C15—N3	117.3 (3)
C3—C4—C5	118.3 (3)	C20—C15—N3	123.5 (3)
C3—C4—H4	120.8	C17—C16—C15	121.0 (3)
C5—C4—H4	120.8	C17—C16—H16	119.5
N1—C5—C4	122.8 (3)	C15—C16—H16	119.5
N1—C5—C14	116.4 (2)	C16—C17—C18	120.2 (4)
C4—C5—C14	120.8 (3)	C16—C17—H17	119.9
O1—C6—N2	125.0 (3)	C18—C17—H17	119.9
O1—C6—C1	121.5 (3)	C19—C18—C17	119.3 (3)
N2—C6—C1	113.5 (2)	C19—C18—C21	121.2 (4)
C12—C7—C8	119.1 (3)	C17—C18—C21	119.4 (4)
C12—C7—N2	118.1 (3)	C18—C19—C20	121.1 (3)
C8—C7—N2	122.7 (3)	C18—C19—H19	119.4
C9—C8—C7	119.5 (3)	C20—C19—H19	119.4
C9—C8—H8	120.3	C19—C20—C15	119.1 (3)
C7—C8—H8	120.3	C19—C20—H20	120.4
C10—C9—C8	121.2 (3)	C15—C20—H20	120.4
C10—C9—H9	119.4	F5—C21—F4'	118.5 (12)
C8—C9—H9	119.4	F5—C21—F4	111.1 (7)
C9—C10—C11	119.6 (3)	F4'—C21—F4	78.5 (7)
C9—C10—C13	121.1 (3)	F5—C21—F6'	23.6 (8)
C11—C10—C13	119.3 (4)	F4'—C21—F6'	106.3 (8)
C12—C11—C10	119.9 (3)	F4—C21—F6'	131.1 (9)
C12—C11—H11	120.0	F5—C21—F5'	79.7 (7)
C10—C11—H11	120.0	F4'—C21—F5'	108.5 (7)
C11—C12—C7	120.7 (3)	F4—C21—F5'	35.8 (6)
C11—C12—H12	119.7	F6'—C21—F5'	102.9 (7)
C7—C12—H12	119.7	F5—C21—F6	105.8 (7)
F2—C13—F1	108.7 (6)	F4'—C21—F6	24.6 (7)
F2—C13—F3'	29.8 (7)	F4—C21—F6	102.8 (6)
F1—C13—F3'	127.3 (8)	F6'—C21—F6	87.6 (10)
F2—C13—F2'	128.5 (8)	F5'—C21—F6	129.5 (7)
F1—C13—F2'	79.0 (7)	F5—C21—C18	114.4 (8)
F3'—C13—F2'	105.0 (7)	F4'—C21—C18	116.3 (8)
F2—C13—F3	106.7 (6)	F4—C21—C18	112.5 (6)
F1—C13—F3	105.2 (6)	F6'—C21—C18	108.3 (8)
F3'—C13—F3	78.8 (7)	F5'—C21—C18	113.5 (6)
F2'—C13—F3	29.3 (5)	F6—C21—C18	109.3 (6)
F2—C13—F1'	79.9 (7)	C5—N1—C1	118.1 (2)
F1—C13—F1'	31.5 (6)	C6—N2—C7	129.3 (3)
F3'—C13—F1'	105.0 (7)	C6—N2—H2A	117 (2)
F2'—C13—F1'	106.9 (7)	C7—N2—H2A	114 (2)
F3—C13—F1'	127.2 (8)	C14—N3—C15	130.2 (3)
F2—C13—C10	110.7 (6)	C14—N3—H3A	115 (2)
F1—C13—C10	112.1 (6)	C15—N3—H3A	115 (2)
			
N1—C1—C2—C3	−1.6 (5)	C4—C5—C14—N3	−160.5 (3)
C6—C1—C2—C3	179.6 (3)	C20—C15—C16—C17	1.3 (5)
C1—C2—C3—C4	0.5 (5)	N3—C15—C16—C17	−179.7 (3)
C2—C3—C4—C5	0.9 (5)	C15—C16—C17—C18	−1.7 (6)
C3—C4—C5—N1	−1.5 (5)	C16—C17—C18—C19	0.9 (6)
C3—C4—C5—C14	177.6 (3)	C16—C17—C18—C21	178.5 (4)
N1—C1—C6—O1	−159.9 (3)	C17—C18—C19—C20	0.3 (6)
C2—C1—C6—O1	19.0 (5)	C21—C18—C19—C20	−177.2 (4)
N1—C1—C6—N2	18.9 (4)	C18—C19—C20—C15	−0.7 (5)
C2—C1—C6—N2	−162.2 (3)	C16—C15—C20—C19	0.0 (5)
C12—C7—C8—C9	−2.3 (5)	N3—C15—C20—C19	−179.0 (3)
N2—C7—C8—C9	178.7 (3)	C19—C18—C21—F5	96.4 (7)
C7—C8—C9—C10	0.1 (5)	C17—C18—C21—F5	−81.2 (7)
C8—C9—C10—C11	1.3 (5)	C19—C18—C21—F4'	−47.7 (8)
C8—C9—C10—C13	−175.6 (3)	C17—C18—C21—F4'	134.7 (8)
C9—C10—C11—C12	−0.6 (5)	C19—C18—C21—F4	−135.6 (6)
C13—C10—C11—C12	176.3 (4)	C17—C18—C21—F4	46.8 (7)
C10—C11—C12—C7	−1.5 (5)	C19—C18—C21—F6'	71.8 (8)
C8—C7—C12—C11	3.0 (5)	C17—C18—C21—F6'	−105.7 (7)
N2—C7—C12—C11	−178.0 (3)	C19—C18—C21—F5'	−174.6 (6)
C9—C10—C13—F2	75.5 (6)	C17—C18—C21—F5'	7.8 (7)
C11—C10—C13—F2	−101.4 (6)	C19—C18—C21—F6	−22.1 (7)
C9—C10—C13—F1	−46.1 (7)	C17—C18—C21—F6	160.3 (6)
C11—C10—C13—F1	137.0 (6)	C4—C5—N1—C1	0.6 (4)
C9—C10—C13—F3'	107.6 (7)	C14—C5—N1—C1	−178.6 (2)
C11—C10—C13—F3'	−69.4 (7)	C2—C1—N1—C5	1.0 (4)
C9—C10—C13—F2'	−133.0 (6)	C6—C1—N1—C5	179.9 (2)
C11—C10—C13—F2'	50.1 (7)	O1—C6—N2—C7	2.6 (5)
C9—C10—C13—F3	−164.8 (6)	C1—C6—N2—C7	−176.2 (3)
C11—C10—C13—F3	18.2 (7)	C12—C7—N2—C6	159.3 (3)
C9—C10—C13—F1'	−11.9 (7)	C8—C7—N2—C6	−21.7 (5)
C11—C10—C13—F1'	171.2 (6)	O2—C14—N3—C15	3.4 (5)
N1—C5—C14—O2	−161.2 (3)	C5—C14—N3—C15	−176.4 (3)
C4—C5—C14—O2	19.7 (4)	C16—C15—N3—C14	161.7 (3)
N1—C5—C14—N3	18.6 (4)	C20—C15—N3—C14	−19.3 (5)

### Hydrogen-bond geometry (Å, °)

**Table:**

*D*—H···*A*	*D*—H	H···*A*	*D*···*A*	*D*—H···*A*
N2—H2A···O1^i^	0.84 (3)	2.58 (3)	3.303 (4)	144 (3)
N3—H3A···O2^ii^	0.85 (4)	2.36 (4)	3.051 (3)	139 (3)

Symmetry codes: (i) *x*, −*y*+1/2, *z*−1/2; (ii) *x*, −*y*+1/2, *z*+1/2.
